# RETRACTED: Hosni Mahmoud, H.A.; Alabdulkreem, E. Bidirectional Neural Network Model for Glaucoma Progression Prediction. *J. Pers. Med.* 2023, *13*, 390

**DOI:** 10.3390/jpm14020148

**Published:** 2024-01-29

**Authors:** Hanan A. Hosni Mahmoud, Eatedal Alabdulkreem

**Affiliations:** Department of Computer Sciences, College of Computer and Information Sciences, Princess Nourah Bint Abdulrahman University, P.O. Box 84428, Riyadh 11671, Saudi Arabia; eaalabdulkareem@pnu.edu.sa

The *Journal of Personalized Medicine* retracts the article Bidirectional Neural Network Model for Glaucoma Progression Prediction [1].

Following publication, concerns were brought to the attention of the Editorial Office regarding a significant overlap with a manuscript under review at another publisher [2].

Adhering to our complaints procedure, an investigation was conducted by the Editorial Office and Editorial Board, which confirmed the extensive and dispersed nature of the overlap and the relative timelines of the submission of the two articles. Consequently, the Editorial Office has decided to retract this publication [1] in accordance with MDPI’s retraction policy (https://www.mdpi.com/ethics#_bookmark30).

This retraction was approved by the Editor-in-Chief of the *Journal of Personalized Medicine*.

The authors did not agree to this retraction.
